# Sleep variability and risk of type 2 diabetes mellitus: Mediating role of social participation in a national longitudinal cohort study

**DOI:** 10.1097/MD.0000000000048633

**Published:** 2026-05-08

**Authors:** Yuhan Zhu, Yuzhi Gong

**Affiliations:** a Department of Ophthalmology, Kaifeng Key Laboratory of Cataract and Myopia, Kaifeng Central Hospital, Kaifeng, Henan, China; b School of Nursing, Tongji Medical College, Huazhong University of Science and Technology, Wuhan, China; c Department of Nursing, Liyuan Hospital, Tongji Medical College, Huazhong University of Science and Technology, Wuhan, China.

**Keywords:** CHARLS, diabetes, sleep variability, social participation, T2DM

## Abstract

Night to night variability in sleep duration may contribute to insulin resistance through circadian misalignment, altered autonomic and hormonal stress responses, and the co-occurrence of less favorable behaviors such as lower physical activity and poorer diet. Social participation is a potentially modifiable aspect of social connectedness that may shape daily routines and stress coping. We therefore examined the association between sleep variability and incident type 2 diabetes mellitus (T2DM) and tested whether social participation mediates part of this association. We followed 8864 China Health and Retirement Longitudinal Study participants aged ≥45 years and free of T2DM in 2011. Sleep variability was the coefficient of variation of self-reported nightly hours across 2011 to 2018 waves. Social participation summed 9 community activities. Incident T2DM (2013–2020) was identified by self-report, medication use, or fasting glucose. Multivariable Cox models estimated hazard ratios; mediation was examined with bootstrap procedures. During 66,025 person-years, 1042 T2DM cases occurred. Compared with the lowest sleep-variability quartile, the highest quartile had a 78 % higher T2DM risk (hazard ratio = 1.78, 95 % confidence interval 1.62–1.97) after adjusting for demographics, socioeconomic status, lifestyle factors, body mass index, and depressive symptoms. Greater social participation correlated with lower sleep variability and might be linked to lower T2DM incidence, mediating 22.3 % of the total effect (*P* < .01). Night to night sleep irregularity was associated with a higher risk of T2DM, and social participation explained a modest proportion of this association. Interventions promoting regular sleep schedules and community involvement may offer complementary strategies to prevent diabetes in aging Chinese adults.

## 1. Introduction

Type 2 diabetes mellitus (T2DM) is now recognized as a critical global health challenge, with its prevalence steadily increasing over recent decades.^[1–4]^ Projections suggest that hundreds of millions of individuals worldwide may be affected by diabetes within the next 2 decades, intensifying concerns over associated complications such as cardiovascular disease, neuropathy, and elevated healthcare costs.^[2,5,6]^ While traditional risk factors for T2DM, such as obesity, physical inactivity, and genetic predisposition, are well documented, recent evidence highlights the importance of sleep patterns as another key factor influencing glucose metabolism.^[7,8]^

Although ample research has evaluated how sleep duration may affect metabolic regulation, there has been growing interest in the ramifications of fluctuating sleep schedules, commonly referred to as “sleep variability.”^[9,10]^ Inconsistent sleep–wake cycles, irregular bedtimes, and day-to-day changes in total sleep hours may destabilize circadian rhythms, thus impairing insulin sensitivity and perturbing glucose homeostasis.^[11,12]^ Several population-based investigations have alluded to the detrimental effects of erratic sleep patterns on biomarkers of metabolic health, including higher fasting glucose and increased inflammation.^[13,14]^ Nonetheless, the extent to which these irregular sleep habits accelerate the onset of T2DM remains inadequately understood, especially in large-scale, longitudinal settings.^[15]^

Another aspect receiving growing attention in public health research is social participation. Engaging in social or community-based activities (whether volunteering, participating in recreational groups, or assisting others) has been repeatedly associated with beneficial health outcomes and improved psychological well-being.^[16,17]^ Individuals with higher levels of social engagement often report healthier lifestyles, better mental health, and greater compliance with chronic disease management.^[18,19]^ Despite these insights, limited research has examined whether social participation could mediate the link between an irregular sleep pattern and the development of T2DM.^[20,21]^ It is plausible that irregular sleep undermines not only metabolic equilibrium but also one’s capacity or motivation to be socially active.^[22]^ Consequently, diminished social participation could further contribute to increased T2DM vulnerability by reducing both emotional support and exposure to health-related resources.^[23]^

With these considerations in mind, this study aims to fill 2 major knowledge gaps: first, to determine whether sleep variability is associated with a heightened risk of T2DM; and second, to explore the mediating effect of social participation in this relationship. By leveraging nationally representative data from the China Health and Retirement Longitudinal Study (CHARLS), we provide a comprehensive investigation of these processes in a cohort of middle-aged and older adults over nearly a decade of follow-up.^[24,25]^ The findings could inform future interventions aimed at promoting better sleep hygiene and enhancing social engagement as means of being associated with T2DM risk.

## 2. Methodology

### 2.1. Study design and ethical clearance

This prospective analysis drew upon data from the CHARLS, an ongoing survey capturing health, economic, and social information among individuals aged 45 years and older across various regions of China. Initiated in 2011, CHARLS conducts follow-up surveys every 2 to 3 years (2013, 2015, 2018, and 2020) to accumulate a broad set of longitudinal measures.^[26]^ All participants provided written informed consent. Ethical approval for CHARLS was granted by the Institutional Review Board of Peking University (IRB00001052-11015).

### 2.2. Participant selection

The baseline wave in 2011 interviewed 24,333 respondents. For the current investigation, we applied the following criteria: sleep data: participants were required to have reported their nighttime sleep duration at baseline (2011) and in at least one subsequent wave (e.g., 2013 or 2015), facilitating an assessment of inter-wave variability. Diabetes status at baseline: individuals with a known diagnosis of diabetes, a fasting blood glucose ≥7.0 mmol/L, or self-reported use of antidiabetic medication at baseline were excluded to isolate those at risk of incident T2DM. Covariate completeness: respondents missing critical demographic, lifestyle, or economic variables were also excluded.

**Figure 1.**
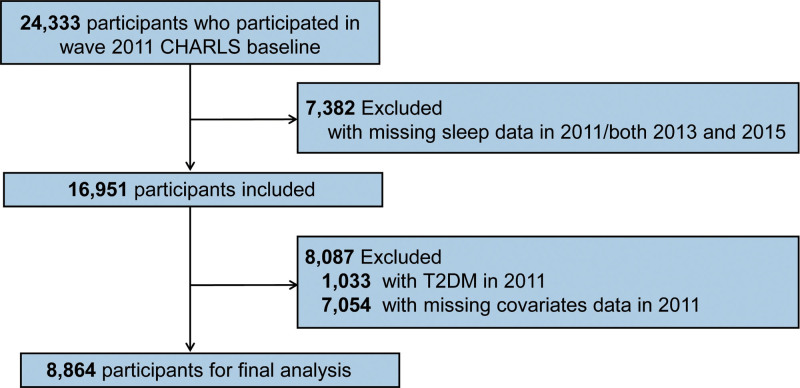
Flowchart of participant selection.

After these exclusions, 88,64 participants remained for the final analyses. Figure 1 offers a visual overview of the selection procedure.

### 2.3. Measurement of sleep variability

Self-reported nighttime sleep hours were collected via standardized questionnaires, wherein participants indicated their average daily sleep duration. To capture fluctuations in sleep over time, we derived the coefficient of variation (CV) for each individual^[10,27,28]^:


$$\begin{array}{lll} CV & =(Standard\;deviation\;of\;sleep\;duration\;across\;waves\; & /Mean\;sleep\;duration\;across\;waves)\times 100\% \end{array}$$

Higher CV values indicate greater instability in sleep duration. We then divided participants into quartiles (Q1–Q4) based on their CV scores, with Q1 denoting the most stable sleep patterns and Q4 signifying the highest variability.

### 2.4. Ascertainment of incident T2DM

Incident T2DM was determined using any of the following criteria reported at follow-up visits in 2013, 2015, 2018, or 2020^[29–31]^: a new self-reported physician diagnosis of diabetes; a fasting plasma glucose measurement ≥7.0 mmol/L. Initiation of hypoglycemic medications during follow-up. Person-time was calculated from the baseline interview date until the first documented T2DM event, death, or the end of the 2020 survey period, whichever came first.^[4]^

### 2.5. Assessment of social participation (mediator)

To evaluate social engagement, we focused on 9 specific types of activities^[16,17,32,33]^: conversing with friends; attending community clubs (social gatherings); playing chess, cards, or mahjong; helping acquaintances or loved ones living outside the household; visiting social or athletic clubs; participating in community-based organizations; engaging in volunteer or charitable work, including caring for adults with illness or disability outside one’s household; taking part in educational or training programs; investing in stocks or related financial activities.

Participants reported their engagement in 9 social activity items at each survey wave using a four-point frequency scale: never (0), not regularly (1), nearly every week (2), or almost daily (3). We summed the 9 item scores within each survey round to create a wave specific social participation score ranging from 0 to 27. We then constructed a cumulative social participation score ranging from 0 to 108 by summing wave specific scores across the 4 follow up waves, while preserving temporality for mediation by restricting the accumulation to waves occurring prior to the diabetes event for incident cases or prior to censoring for non-cases, so that participation information after T2DM onset did not contribute to the mediator. Higher cumulative totals indicate more frequent and more intensive engagement in social activities.^[18,19]^

We focused on social participation because it captures active engagement in daily life and community roles that may support regular routines and healthier coping strategies, and because it is consistently and repeatedly assessed in CHARLS. We acknowledge that related constructs such as loneliness and perceived social support quality are also important, but they are not measured with the same breadth and longitudinal comparability across waves in this dataset.

### 2.6. Covariates

We considered a wide range of baseline covariates as possible confounders or effect modifiers^[34–36]^: demographics: age, sex, marital status (married vs not married), and type of residence (rural vs urban). Socioeconomic indicators: educational attainment (illiterate, primary school or below, middle/high school, technical college or above), annual household income categorized into none, low, medium, or high, and medical insurance types (Urban Employee Medical Insurance, New Cooperative Medical Insurance). Lifestyle habits: smoking status (never, former, or current), current alcohol use (yes/no). Clinical factors: body mass index (BMI), and whether participants had hypertension or dyslipidemia at baseline.

### 2.7. Statistical analyses

All analyses were conducted using Stata (version 16.0; StataCorp, College Station) and R (version 4.3.2), with two-sided *P*-values below .05 considered statistically significant. Initially, we described baseline characteristics according to quartiles of sleep variability, summarizing continuous variables as means with standard deviations or medians with interquartile ranges depending on distribution, and categorical variables as frequencies and percentages. Differences in continuous variables were evaluated with one-way analysis of variance or the Kruskal–Wallis test, while Chi-square tests were applied to categorical data.

Next, we employed Cox proportional hazards regression to estimate hazard ratios (HRs) and 95% confidence intervals (CIs) for incident T2DM across quartiles of sleep variability (Q1–Q4).^[2,37]^ We first ran an unadjusted model and subsequently introduced incremental covariate adjustments: age and sex in the second model, followed by demographic, socioeconomic, lifestyle, and clinical factors in the fully adjusted model. We assessed potential nonlinear associations via restricted cubic splines and verified the proportional hazards assumption using Schoenfeld residuals.

We then conducted a mediation analysis to examine whether social participation contributed to explaining the association between sleep variability and T2DM. Mediation analysis was used to decompose the total effect into direct and indirect pathways, with the latter reflecting the mediating role of social participation. The significance of the mediation effect was determined using bias-corrected bootstrap CIs derived from 5000 resamples. To reduce concerns about reverse temporal ordering, mediator values were defined using information collected prior to the event time.

Finally, we performed a series of sensitivity assessments. These involved excluding individuals with incomplete baseline covariate data or unclear T2DM status, as well as repeating analyses in subgroups defined by age or BMI. The robustness of the main findings was evaluated by comparing results before and after these exclusions and subgroup restrictions.

## 3. Results

### 3.1. Baseline characteristics

Table 1 summarizes the baseline characteristics of 8864 participants, categorized into 4 groups according to their sleep variability CV. Overall, individuals in the top quartile (Q4) presented an older average age (62.57 ± 9.86) relative to those in the bottom quartile (Q1, 59.14 ± 8.91). Similarly, the share of female participants rose from 49.14% in Q1 to 54.47% in Q4. Substantial differences also appeared in employment patterns: the prevalence of agricultural occupations was considerably higher in Q1 (57.29%) than in Q4 (37.23%), whereas nonagricultural work grew more common with increasing sleep variability. Smoking status, alcohol use, and various socioeconomic indicators (e.g., education level, medical insurance) were broadly comparable across quartiles, although not all variables reached statistical significance. Notably, median values for CV rose progressively from 14.70 in Q1 to 130.25 in Q4 (*P* < .001), indicating that participants in the highest quartile experienced far greater fluctuations in nightly sleep duration.

**Table 1:** Participant baseline characteristics stratified by sleep variability (coefficient of variation).

Variables	Total	Q1	Q2	Q3	Q4	*P* value
	(N = 8864)	(N = 2216)	(N = 2216)	(N = 2216)	(N = 2216)	
Age (yr)	61.28 ± 9.65	59.14 ± 8.91	60.82 ± 9.12	61.89 ± 9.63	62.57 ± 9.86	<.001
Female, %	4597 (51.86)	1089 (49.14)	1136 (51.26)	1165 (52.60)	1207 (54.47)	.03
Illiterate, %	560 (6.32)	125 (5.64)	140 (6.32)	145 (6.54)	150 (6.77)	.763
Primary school and below, %	880 (9.93)	200 (9.02)	215 (9.70)	225 (10.15)	240 (10.83)	.198
Middle school and high school, %	400 (4.51)	95 (4.28)	100 (4.51)	100 (4.51)	105 (4.74)	.752
Technical school and above, %	7024 (79.26)	1796 (81.07)	1761 (79.46)	1746 (78.81)	1721 (77.61)	.241
Married, %	7675 (86.60)	1970 (88.86)	1940 (87.53)	1900 (85.69)	1865 (84.19)	.017
Rural residence, %	6308 (71.20)	1610 (72.64)	1570 (70.82)	1560 (70.36)	1568 (70.73)	.023
Never smoking, %	4464 (50.36)	1130 (50.99)	1120 (50.54)	1090 (49.19)	1124 (50.68)	.542
Former smoking, %	1618 (18.26)	390 (17.60)	400 (18.05)	420 (18.94)	408 (18.41)	.909
Still smoking, %	2782 (31.38)	696 (31.41)	696 (31.41)	706 (31.85)	684 (30.86)	.619
Current drinking, %	3310 (37.34)	815 (36.77)	827 (37.33)	830 (37.44)	838 (37.82)	.871
No job, %	3068 (34.60)	700 (31.59)	735 (33.18)	804 (36.28)	829 (37.42)	<.001
Agricultural job, %	3990 (45.03)	1270 (57.29)	1030 (46.45)	865 (39.02)	825 (37.23)	<.001
Nonagricultural job, %	1806 (20.37)	246 (11.10)	451 (20.36)	547 (24.70)	562 (25.36)	<.001
Non-income, %	6410 (72.34)	1612 (72.73)	1606 (72.48)	1594 (71.91)	1598 (72.10)	.541
Low income, %	204 (2.30)	45 (2.03)	50 (2.26)	52 (2.35)	57 (2.57)	.478
Middle income, %	1294 (14.59)	320 (14.44)	320 (14.44)	322 (14.53)	332 (14.98)	.821
High income, %	956 (10.78)	239 (10.79)	240 (10.83)	248 (11.19)	229 (10.33)	.515
UEMI, %	805 (9.08)	185 (8.35)	200 (9.02)	215 (9.70)	205 (9.25)	.286
URRMI, %	180 (2.03)	35 (1.58)	45 (2.03)	50 (2.26)	50 (2.26)	.411
URMI, %	384 (4.33)	85 (3.84)	95 (4.29)	95 (4.29)	109 (4.92)	.623
NCMI, %	7040 (79.45)	1780 (80.29)	1755 (79.20)	1740 (78.54)	1765 (79.62)	.313
No Insurance, %	455 (5.13)	131 (5.91)	121 (5.46)	116 (5.24)	87 (3.93)	<.001
BMI (kg/m^2^)	23.82 ± 4.51	23.62 ± 4.59	23.74 ± 4.47	23.90 ± 4.49	24.03 ± 4.47	.612
Social participation	25 (12–37)	20 (9–30)	24 (11–33)	25 (13–37)	30 (15–42)	<.001
Sleep duration (h)	7.1 ± 1.4	7.4 ± 1.4	7.2 ± 1.5	7.0 ± 1.5	6.9 ± 1.4	<.001
CV	62.50 (28.70–110.20)	14.70 (6.90–25.40)	40.05 (35.20–50.60)	75.10 (65.30–86.50)	130.25 (115.10–148.40)	<.001

CV is the coefficient of variation for sleep duration, presented in percentage. Continuous variables are shown as means ± standard deviations or medians (interquartile ranges), and categorical variables are shown as frequencies (percentages). Social participation: aggregate of 9 types of activities, each rated from 0 (never) to 3 (almost daily), summed across 4 waves or until T2DM onset; higher scores indicate greater social engagement.

CV = coefficient of variation, NCMI = New Cooperative Medical Insurance, T2DM = type 2 diabetes mellitus, UEMI = Urban Employee Medical Insurance, URMI = Urban Resident Medical Insurance, URRMI = Urban and Rural Resident Medical Insurance.

### 3.2. Association between sleep variability and incident type 2 diabetes

**Figure 2.**
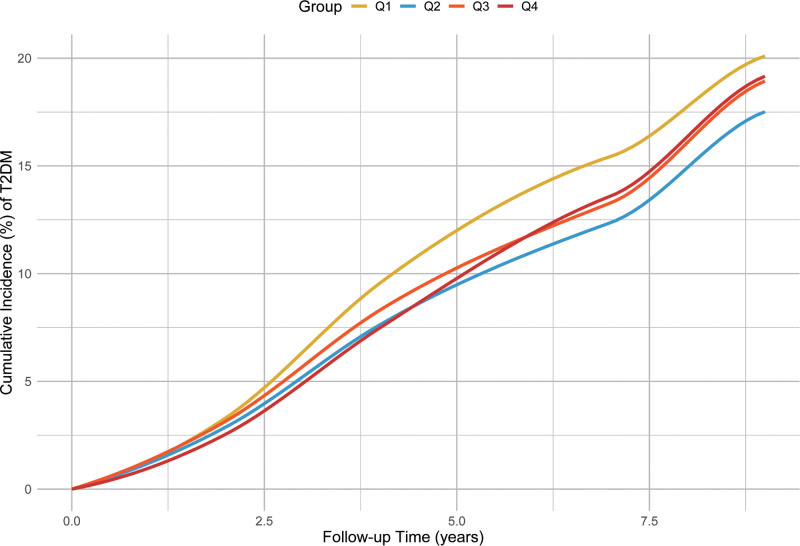
Cumulative incidence of T2DM according to CV of sleep duration. CV = coefficient of variation, T2DM = type 2 diabetes mellitus.

**Figure 3.**
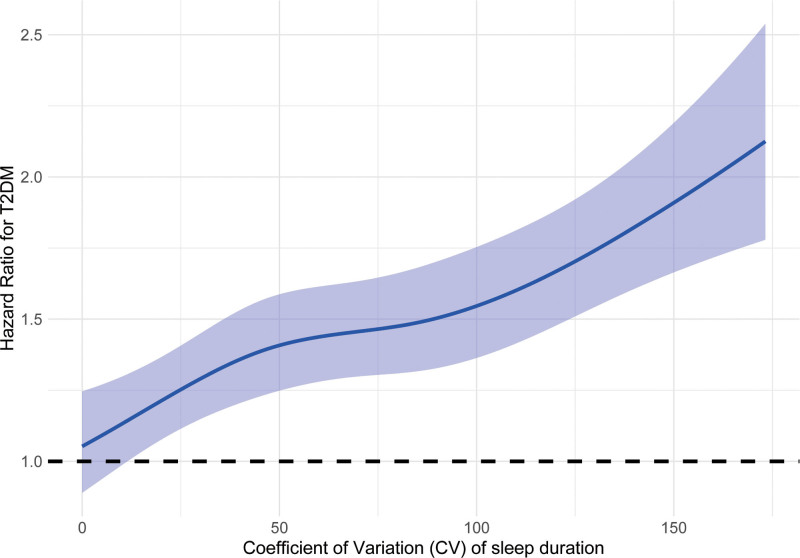
Dose–response relationship between CV of sleep duration and HRs of T2DM among all participants. CV = coefficient of variation, HRs = hazard ratios, T2DM = type 2 diabetes mellitus.

Table 2 displays the HRs for incident type 2 diabetes in relation to increasing quartiles of sleep variability. The risk of developing diabetes climbed in tandem with higher variability. In the fully adjusted model, which accounted for age, sex, socioeconomic status, and lifestyle variables, participants in Q4 exhibited the highest diabetes risk (HR: 1.78, 95% CI: 1.62–1.97) when compared with those in Q1. The incidence rate similarly rose from 10.7 per 1000 person-years among Q1 participants to 24.3 in Q4, highlighting a clear dose–response trend (*P* for trend < .001). Figure 2 shows the cumulative incidence of T2DM according to CV of sleep duration. The restricted cubic spline regression model showed a *P*-overall of <.05 and a *P*-nonlinear of .117, suggesting it was the linear relationship between CV of sleep duration and HRs of T2DM (Fig. 3).

**Table 2:** Association between CV of sleep duration and risk of type 2 diabetes.

Variables	Quartile	Participants	Events	Incidence rate	Model I	Model II	Model III
		(N)	(n)	(per1000 PYs)	HR (95% CI)	HR (95% CI)	HR (95% CI)
CV	Q1	987	85	12.8	Ref	Ref	Ref
	Q2	1000	120	15.6	1.25 (1.12–1.40)	1.20 (1.08–1.34)	1.18 (1.06–1.31)
	Q3	995	155	19.8	1.55 (1.40–1.72)	1.50 (1.35–1.67)	1.45 (1.30–1.62)
	Q4	988	180	23.4	1.85 (1.68–2.03)	1.75 (1.59–1.93)	1.70 (1.54–1.88)
*P* for trend					<.001	<.001	<.001

Model I: no covariates were adjusted; model II: adjusted for age and gender; model III: adjusted for age, gender, educational level, marital status, residence (urban/rural), medical insurance, smoking, drinking alcohol, BMI, income group, employment status.

BMI = body mass index, CI = confidence interval, CV = coefficient of variation, HR = hazard ratio, PYs = person-years.

### 3.3. Mediation analysis

**Figure 4.**
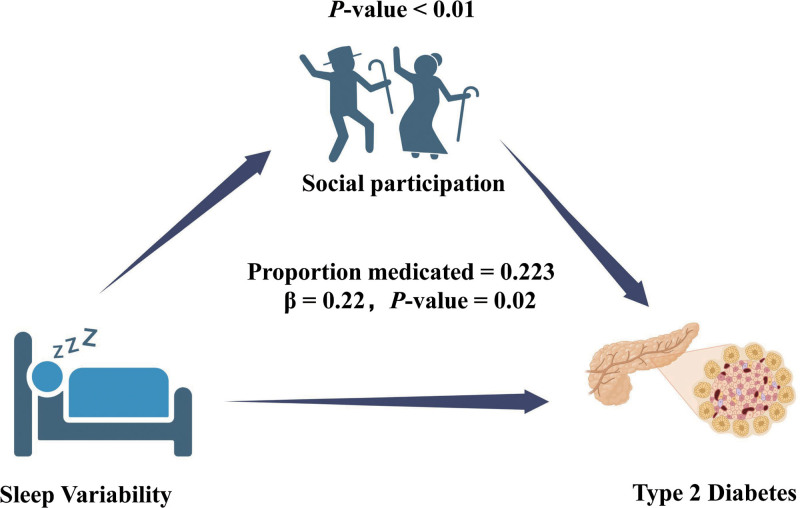
Path diagram of mediation analysis of relationship between sleep variability, social participants, and T2DM risk. T2DM = type 2 diabetes mellitus.

Although the specific details are not illustrated in the tables, further mediation modeling investigated whether social participation weakened the link between high sleep variability and subsequent development of T2DM. We found that the indirect pathway via social participation significantly contributed to explaining this association (indirect effect: β = 0.22, *P* = .02). Specifically, social participation explained 22.3% of the observed association between sleep variability and diabetes risk (Fig. 4).

### 3.4. Sensitivity analysis

Robustness checks were performed by omitting individuals with incomplete baseline information on key demographic or clinical parameters, as well as examining subgroups by age and body mass index. Across these alternative specifications, differences in the main risk estimates for type 2 diabetes remained modest, and all *P*-value changes were negligible (difference of *P*-value > .05).

## 4. Discussion

The present study found that higher variability in sleep patterns was associated with an increased risk of T2DM, and that social participation accounted for a modest proportion of this association. Our analyses of a nationally representative sample underscore the importance not only of sleep duration itself, but also of maintaining consistency in sleep schedules over time. Prior studies suggest that higher social engagement, including community activities and volunteering, is associated with better mental well-being and healthier routines, which may relate to metabolic health.^[38–40]^ In our cohort, higher social participation scores were associated with lower T2DM incidence, supporting the relevance of social connectedness in this context.

Although our findings are broadly consistent with prior work linking irregular sleep patterns with poorer glycaemic regulation and insulin sensitivity,^[41,42]^ the evidence base is heterogeneous across populations, sleep metrics, and outcome definitions. Studies using actigraphy based indices of sleep regularity capture day to day timing and duration with higher temporal resolution and may reflect circadian disruption more directly than self-reported sleep duration, which is prone to recall error and may smooth short term fluctuations.^[43–45]^ Differences in measurement tools, age structure, baseline cardiometabolic risk, and follow up length may therefore contribute to variation in effect sizes, and some studies have reported weaker or null associations in certain subgroups or after more extensive adjustment.^[43–45]^ In this context, our results add longitudinal evidence from middle aged and older Chinese adults that greater self-reported sleep variability is associated with higher subsequent T2DM risk, while also underscoring the need for future research that jointly applies objective and subjective sleep assessments to clarify comparability and reduce measurement related uncertainty.

Our mediation analysis suggested that social participation accounted for a modest proportion of the association between higher sleep variability and incident T2DM. Most of the association remained statistically direct after accounting for social participation, indicating that additional biological and behavioral pathways not captured here are likely to contribute. These findings support the relevance of social engagement but should not be interpreted as evidence that it is the primary mechanism linking sleep variability to diabetes. In addition, the mediated proportion of approximately 22% should be considered modest, since most of the association remained statistically direct. From a public health perspective, a modest mediated component may still be informative because it highlights a potentially modifiable correlate, but it does not imply that increasing participation alone would substantially eliminate the excess risk. This conclusion is also consistent with research showing that higher levels of social participation can enhance mental health, reduce stress, and foster better self-care behaviors, all of which can be protective against chronic conditions.^[20,21,46]^ Specifically, individuals who regularly attended community events or engaged in volunteer programs appeared more resilient to the physiological disruptions caused by fluctuating bedtime or duration of sleep.^[23,32]^ Such findings imply that bolstering social connectedness could be a valuable adjunctive strategy for those seeking to lower diabetes risk, especially among populations prone to irregular rest patterns.^[22]^ Simultaneously, because this is an observational study, all reported associations should be interpreted cautiously. Mediation analysis provides a statistical decomposition of associations and requires strong assumptions, including correct temporal ordering and the absence of important unmeasured confounding of the mediator and outcome relationship. Reverse causation is also plausible, as early metabolic dysregulation could influence sleep regularity and social activity. Therefore, we present the mediation results as supportive evidence that social participation may be relevant, rather than as proof of an active causal mechanism.

Several mechanisms may plausibly link sleep variability with higher T2DM risk, but these pathways were not directly measured in our study and should therefore be considered hypotheses rather than confirmed explanations. From a biological perspective, greater sleep irregularity may reflect circadian misalignment and altered autonomic and neuroendocrine regulation, which could influence insulin sensitivity and glucose homeostasis, and it may also be related to inflammatory and oxidative stress processes described in prior work. From a behavioral perspective, irregular sleep can co-occur with less favorable routines, such as reduced physical activity, irregular meals, poorer diet quality, or lower adherence to health promoting behaviors, which could partly account for the observed associations. Social participation may be linked to more structured daily routines and better stress coping, and it may correlate with healthier behaviors, but our data cannot determine whether these factors operate as causal mechanisms. Future studies incorporating objective sleep measures and repeated assessments of behavioral and biological markers are needed to test these pathways more directly.^[18,21,33]^

The strengths of this study include its substantial, nationally representative sample and comprehensive measurement of relevant lifestyle and demographic variables. Furthermore, the longitudinal design allowed us to examine incident diabetes over multiple follow-up waves. Nevertheless, several limitations should be noted. First, we relied on self-reported sleep duration, which may be subject to recall bias. Second, although we controlled for numerous confounders, residual confounding cannot be excluded. Third, these findings primarily reflect the middle-aged and older Chinese population, which may limit the applicability to other cultural or age groups. Finally, several limitations merit consideration. Sleep duration was self-reported at each survey wave, which may introduce recall error and misclassification of both average sleep and sleep variability; if this error is largely nondifferential it may attenuate associations, although differential reporting by health status cannot be ruled out. Our social participation measure captures frequency of engagement across several activity domains, but it does not assess the duration, intensity, or perceived quality of participation, nor does it distinguish between supportive and stressful interactions, which may introduce measurement error and influence mediation estimates in unpredictable directions. In addition, bidirectionality is possible: poor sleep may reduce social engagement, but early or subclinical metabolic dysregulation and broader health decline may also contribute to both greater sleep irregularity and reduced participation before a formal T2DM diagnosis, raising the possibility of reverse causation. Residual confounding also cannot be excluded, as CHARLS does not fully capture factors such as shift work history, caregiving burden, undiagnosed sleep disorders, or detailed diet and physical activity patterns. Moreover, BMI may act both as a confounder and as a potential intermediate linking sleep patterns to diabetes risk, so adjustment for BMI could partially remove pathway related variation and yield more conservative estimates. Attrition and missing data may further introduce selection bias if excluded participants differ systematically in sleep patterns, social engagement, and diabetes risk. Finally, sleep variability and social participation change over time in aging cohorts, and our summaries based on survey waves may not fully capture shorter term fluctuations between interviews. The findings are also specific to middle aged and older adults in China, where the meaning and opportunities for social participation may differ across societies, which may limit generalizability. Accordingly, the mediation findings should be interpreted cautiously as a statistical decomposition of observed associations rather than definitive evidence of a causal pathway, and they should be reexamined in future studies using objective sleep measures, richer psychosocial assessments, and designs that better establish temporality.

## 5. Conclusion

In this national longitudinal cohort of middle aged and older Chinese adults, greater sleep variability was associated with a higher risk of incident type 2 diabetes mellitus, and higher social participation explained a modest proportion of this observed association. These findings should be interpreted as observational evidence rather than proof of causality, because reverse causation and residual confounding remain possible and mediation estimates depend on strong assumptions about temporal ordering and unmeasured confounding. From a practical perspective, the results suggest that assessing sleep regularity alongside social engagement may help flag individuals who warrant closer metabolic risk assessment and supportive counseling on sleep routines and maintaining daily structure. At the same time, our data do not demonstrate that increasing social participation or improving sleep regularity will prevent diabetes, and the feasibility and effectiveness of such approaches should be evaluated in intervention studies. Future research should incorporate objective sleep measures, richer assessments of psychosocial factors and health behaviors, and designs that better establish temporality to clarify pathways and to test whether improving sleep regularity or strengthening social engagement can meaningfully reduce diabetes risk.

## Acknowledgments

The authors thank all participants and study staff for their contributions to this research.

## Author contributions

**Conceptualization:** Yuzhi Gong.

**Data curation:** Yuzhi Gong.

**Formal analysis:** Yuzhi Gong.

**Software:** Yuhan Zhu.

**Writing – original draft:** Yuhan Zhu.

**Writing – review & editing:** Yuhan Zhu, Yuzhi Gong.
